# Left bundle branch pacing optimized right ventricular resynchronization in a patient with severe right ventricular dilatation

**DOI:** 10.1016/j.hrcr.2026.02.010

**Published:** 2026-02-17

**Authors:** Zhihui Zhao, Lukas Poviser, Jan Mizner, Karol Curila

**Affiliations:** Cardiocenter, Third Faculty of Medicine, Charles University and University Hospital Kralovske Vinohrady, Prague, Czech Republic

**Keywords:** Left bundle branch pacing, Ventricular dyssynchrony, LBBP-optimized RV-CRT, Ultra-high-frequency ECG, Cardiac resynchronization therapy

**Key Teaching Points**

• In patients with marked right ventricular (RV) dilatation, left bundle branch pacing (LBBP) may not fully correct RV activation delay.
• Supplemental RV lateral-wall pacing can further improve RV activation and achieve effective RV resynchronization when LBBP alone is insufficient.
• Ultra-high-frequency electrocardiogram provides a quantitative assessment of ventricular activation and is useful for evaluating dyssynchrony during individualized pacing optimization.
• Combining LBBP with RV lateral-wall pacing represents a feasible LBBP-optimized RV-cardiac resynchronization therapy approach for selected patients with significant RV structural remodeling.

## Introduction

Cardiac resynchronization therapy (CRT) is an effective treatment for patients with left bundle branch block (LBBB) and left ventricular (LV) dyssynchrony. LLB area pacing (LBBAP) has recently emerged as a promising physiological alternative to conventional right ventricular pacing (RVP) and CRT,^1^^,^^2^ but may lead to significant RV dyssynchrony, which might affect clinical outcomes in a specific group of patients.^3^ Here, we present a case of successful RV-CRT achieved by combining LBB pacing (LBBP) with RV lateral wall pacing to restore ventricular synchrony in a patient with intermittent atrioventricular (AV) conduction disease and severe RV dilatation because of an unrepaired atrial septal defect. Quantification of RV dyssynchrony reduction was performed using ultra-high-frequency electrocardiography (UHF-ECG).

## Case presentation

**Figure 1:**
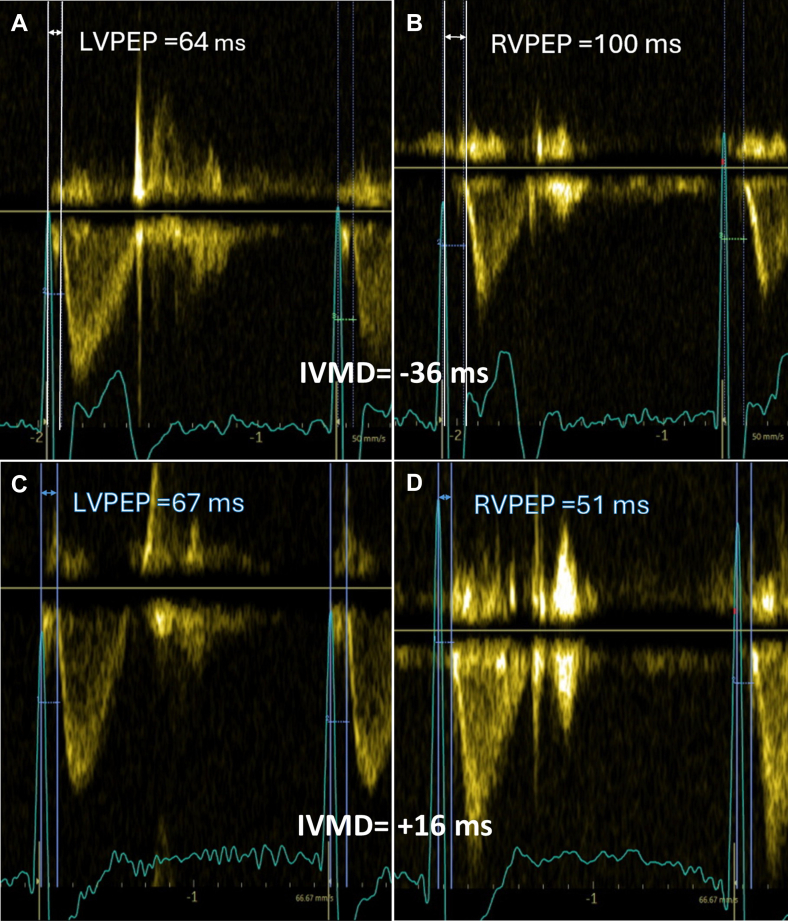
Interventricular mechanical delay (IVMD) before and after right ventricular-cardiac resynchronization therapy implantation. Pulsed-wave Doppler tracings showing LV pre-ejection period (LVPEP) and right ventricular pre-ejection period (RVPEP) with caliper measurements. **A–B:** pre-implantation; **C–D:** post-implantation. IVMD = LVPEP − RVPEP.

An 86-year-old woman with a known atrial septal defect, which had remained untreated at her own decision, was admitted to the hospital because of weakness and reduced exercise tolerance (New York Heart Association III). Her symptoms had been present for more than 6 months, with a marked worsening in a few days before admission. A 12-lead electrocardiogram (ECG) showed atrial fibrillation and right bundle branch block (RBBB) with QRS duration of 164 ms. During ECG monitoring, the heart rate was 50–90 bpm, with episodes of severe bradycardia down to 30 bpm due to intermittent third-degree AV block, observed both during sleep and daytime hours. Transthoracic echocardiography (TTE) showed a secundum-type atrial septal defect with left-to-right shunt (Qp/Qs 3.1), preserved left ventricular function (LVEF 55%), marked dilation of the RV and decreased RV systolic function. Fractional area change (FAC) was 26%; RV free-wall strain −16% and global RV strain −11%. Interventricular mechanical delay (IVMD), measured as the time delay between the opening of the aortic and pulmonary valves, showed −36 ms (negative values indicate delayed RV activation and positive values indicate delayed LV activation)^4^ (Figure 1A and B). Tricuspid regurgitation was mild-to-moderate. The patient consented to permanent pacemaker implantation.

**Figure 2:**
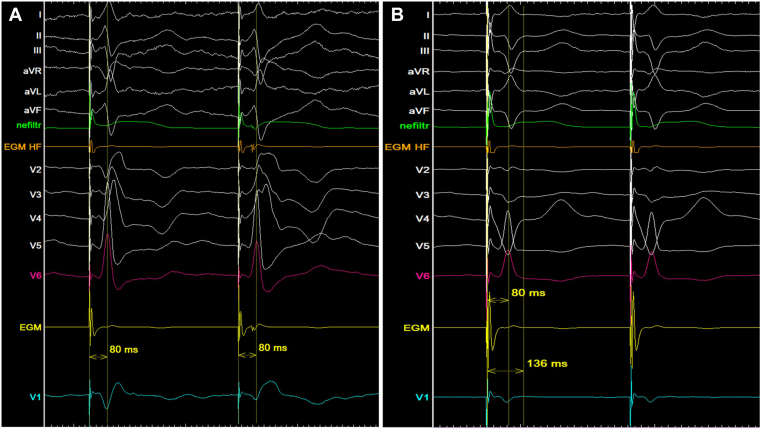
Intra-procedural 12-lead ECG and intracardiac electrograms at 100 mm/s. **(A)** LBB potential recorded during spontaneous rhythm. (A) nsLBBP to sLBBP transition (V6-LVAT 80 ms). **(B)** Combined LBBP + RV lateral-wall pacing (V6-LVAT 80 ms, QRSd 136 ms). ECG = electrocardiogram; LBB = left bundle branch; LVAT = left ventricular activation time; nsLBBP = non-selective left bundle branch pacing; sLBBP = selective LBBP; RV= right ventricle.

During the procedure, LBBP was targeted, and a ventricular lead (Medtronic 3830; 69 cm) was positioned deep in the RV septum, yielding a QR morphology in lead V1 and a left ventricular activation time (LVAT) of 80 ms in lead V6. Subsequently, LBB capture was confirmed by (1) recording of an LBB potential and (2) transitioning from non-selective to selective LBBP during output decrement at 1.6 V (Figure 2A). Absolute pacing threshold 0.5 V at 0.5 ms, R-wave sensing 10.5 mV, and unipolar impedance 734 Ω.

**Figure 3:**
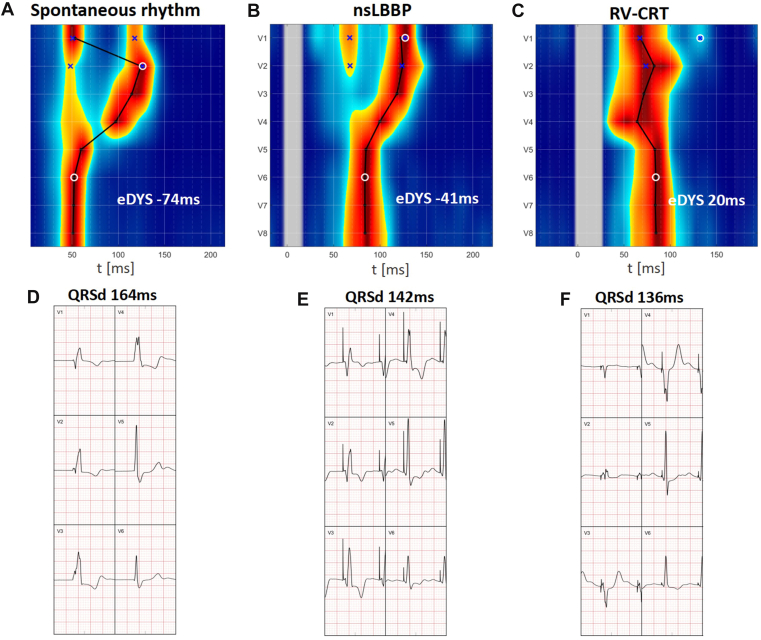
UHF-ECG (**A–C**) and V1-V6 QRS complexes (**D–F**) during the procedure. Spontaneous RBBB rhythm (**A, D**), Non-selective LBBP (**B, E**), and combined LBBP + RV lateral-wall pacing (RV-CRT) (**C, F**).CRT = cardiac resynchronization therapy; LBBP = left bundle branch pacing; RBBB = right bundle branch pacing; RV = right ventricle; UHF-ECG = ultra-high frequency electrocardiogram.

With non-selective LBBP, ventricular dyssynchrony was reduced compared with spontaneous rhythm, and the QRS duration (QRSd) narrowed from 164 ms to 142 ms. However, a significant RV activation delay persisted with electrical dyssynchrony (e-DYS) −41 ms (Figure 3B). Anodal capture was not achievable during a bipolar pacing with an output of 5 V at 0.5 ms.

**Figure 4:**
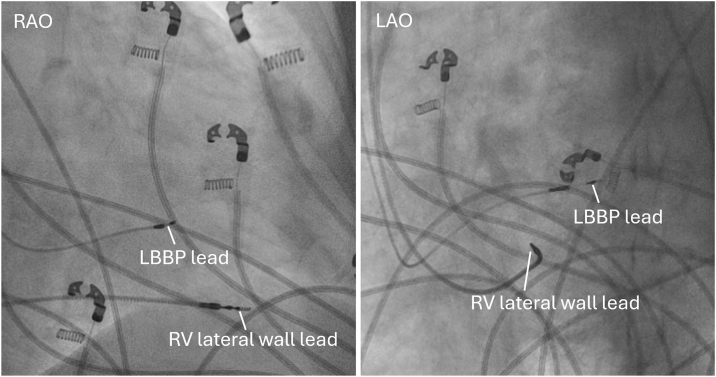
Fluoroscopic views (RAO and LAO) after RV-CRT implantation demonstrating the LBBP lead and the RV lateral-wall lead, *white lines* indicate the lead tips. CRT = cardiac resynchronization therapy; LAO = left anterior oblique; LBBP = left bundle branch pacing; RAO = right anterior oblique; RV = right ventricle.

To reduce RV dyssynchrony, a second ventricular lead (Medtronic 5076; 58cm) was implanted at the distal RV lateral wall (Figure 4). During combined LBBP + RV lateral-wall pacing (RV-CRT), a QS morphology was observed in lead V1, QRSd narrowed slightly to 136 ms (Figure 2B), and UHF-ECG confirmed correction of RV activation delay with a resultant ventricular e-DYS of +20 ms (Figure 3C). The next day the patient was discharged without any procedure-related complications. A biventricular pacemaker was implanted with the LBBP lead connected to RV port and RV lead connected to LV port of the pacemaker. Pacemaker settings were as follows: VVI mode, 65 bpm; VV delay −10 ms (LBBP before the RV lead); unipolar pacing polarity; pacing output 3.5 V at 0.4 ms.

At the 6-week follow-up, pacing parameters remained stable, and the patient reported marked symptomatic improvement, including greater exercise tolerance and absence of dyspnea. TTE showed improved interventricular synchrony, with IVMD decreasing to +16 ms compared with the examination performed before pacemaker implantation (Figure 1C and D). RV systolic function improved, with FAC increasing from 26% to 33%, RV free-wall strain improved from −16% to −18% and global RV strain from −11% to −15%. LVEF remained at 55% and tricuspid regurgitation remained mild-to-moderate, without appreciable change from baseline.

## Discussion

In this report, we present a case of successful RV resynchronization therapy in a patient with markedly dilated RV, spontaneous RBBB, and AV conduction disease. We showed that although direct pacing of the LBB led to a significant reduction in RV activation delay, complete correction was achieved after implantation of an additional lead in the RV lateral wall.

LBBP, as a physiological pacing modality, has recently emerged as a promising alternative to RVP and biventricular pacing.^1^^,^^2^^,^^5^^,^^6^ Although LBBP enables rapid and physiological LV activation, it typically produces some degree of RV activation delay, electrocardiographically manifested as a pseudo-RBBB pattern on the ECG, and a UHF-ECG study has quantified this effect, reporting median e-DYS values of −24 ms during non-selective LBBP and −12 ms during left ventricular septal pacing.^3^^,^^7^ In addition, UHF-ECG–derived interventricular e-DYS has been validated against echocardiographic IVMD.^4^ In patients without structural heart disease, such delayed RV activation resembles native RBBB, which is generally considered benign as it does not result in adverse outcomes.^7^^,^^8^

Nevertheless, chronic pacing-induced electrical and mechanical dyssynchrony is not entirely without risk, as pacing-induced cardiomyopathy has been reported to develop in up to 25% of patients undergoing long-term RVP, although this phenomenon appears less frequent with conduction system pacing.^9^^,^^10^ In contrast to pacemaker recipients without structural heart disease, some patients may be more vulnerable to pacing-related RV dyssynchrony, particularly those with pre-existing RV abnormalities or structural disease, such as congenital heart disease, pulmonary hypertension, or advanced RV failure.^11^ In these patients, pre-existing ventricular remodeling and contractile impairment can amplify the adverse impact of any additional RV activation delay. Under these circumstances, even mild pacing-induced RV dyssynchrony may further impair systolic performance and aggravate heart failure symptoms. Therefore, in patients with marked RV dilation and dysfunction, achieving proper RV resynchronization is crucial to prevent further hemodynamic deterioration.

Our case illustrates this clinical scenario. The patient had long-standing left-to-right shunting across an unrepaired secundum atrial septal defect and spontaneous RBBB, resulting in marked RV dilatation. Although LBBP reduced RV activation delay and improved interventricular synchrony, substantial residual RV dyssynchrony persisted. Therefore, we implanted an additional RV lateral-wall lead, and achieved complete correction of RV activation and further QRS narrowing. After the procedure, the patient reported relief of symptoms, increased exercise capacity, and overall clinical improvement.

Previous studies have also shown that RV-CRT can improve RV activation, contraction efficiency and hemodynamics in selected patients with congenital or right-sided structural heart disease.^12^, ^13^, ^14^ Building on these findings, our case demonstrates an approach that may be described as LBBP-optimized RV-CRT, analogous to left-bundle-optimized CRT (LOT-CRT), in which LBBAP is complemented by an additional coronary-sinus lead to improve LV synchrony. Similarly, in our patient, LBBP improved RV synchrony compared with spontaneous RBBB, but because of residual RV dyssynchrony, implantation of an additional RV lateral-wall lead was necessary.

## Conclusion

LBBP may not fully correct RV dyssynchrony in patients with marked RV remodeling. Supplemental RV lateral-wall pacing can further improve RV synchrony, supporting the feasibility of an LBBP-optimized RV-CRT in such cases.

## Disclosures

Karol Curila has filed a US patent (No: US 11,517,243 B2) on the “Method of electrocardiographic signal processing and apparatus for performing the method” and is a shareholder of VDI Technologies. All other authors declare no conflicts of interest.

## References

[1] Yin L., Wang L., Meng J. (2025). A systematic review and meta-analysis of the impact of left bundle branch area pacing on right ventricular function. Front Cardiovasc Med.

[2] Vijayaraman P., Sharma P.S., Cano Ó. (2023). Comparison of left bundle branch area pacing and biventricular pacing in candidates for resynchronization therapy. J Am Coll Cardiol.

[3] Curila K., Jurak P., Jastrzebski M. (2021). Left bundle branch pacing compared to left ventricular septal myocardial pacing increases interventricular dyssynchrony but accelerates left ventricular lateral wall depolarization. Heart Rhythm.

[4] Mizner J., Beela A., Linkova H. (2025). Electrical and mechanical interventricular dyssynchrony coupling in patients with bradycardia: a UHF-ECG validation trial. Heart Rhythm.

[5] Herweg B., Mumtaz M., Vijayaraman P. (2025). Conduction system pacing for CRT: A physiological alternative. Arrhythm Electrophysiol Rev.

[6] Jastrzębski M., Kiełbasa G., Cano O. (2025). Left bundle branch area pacing vs right ventricular pacing for atrioventricular block: the MELOS RELOADED study. Eur Heart J.

[7] Ozpak E., Demolder A., Kizilkilic S., Calle S., Timmermans F., De Pooter J. (2022). An electrocardiographic characterization of left bundle branch area pacing-induced right ventricular activation delay: A comparison with native right bundle branch block. Front Cardiovasc Med.

[8] Bednarek A., Kiełbasa G., Moskal P. (2024). Left bundle branch area pacing improves right ventricular function and synchrony. Heart Rhythm.

[9] Khurshid S., Frankel D.S. (2023). Pacing-induced cardiomyopathy. Cardiol Clin.

[10] Boyle T.A., Pothineni N.V.K., Austin M. (2024). Incidence and predictors of pacing-induced right ventricular cardiomyopathy. Circ Arrhythm Electrophysiol.

[11] Yeo W.T., Jarman J.W.E., Li W., Gatzoulis M.A., Wong T. (2014). Adverse impact of chronic subpulmonary left ventricular pacing on systemic right ventricular function in patients with congenitally corrected transposition of the great arteries. Int J Cardiol.

[12] Janoušek J., Kovanda J., Ložek M. (2017). Pulmonary right ventricular resynchronization in congenital heart disease: acute improvement in right ventricular mechanics and contraction efficiency. Circ Cardiovasc Imaging.

[13] Sajja A., Ibrahim R., Pernetz M., Lloyd M. (2023). Right ventricular cardiac resynchronization therapy in patients with right ventricular conduction delay and heart failure. Heart Rhythm.

[14] Janoušek J., Kovanda J., Ložek M. (2019). Cardiac resynchronization therapy for treatment of chronic subpulmonary right ventricular dysfunction in congenital heart disease. Circ Arrhythm Electrophysiol.

